# Commentary: Current Status and Issues Regarding Pre-processing of fNIRS Neuroimaging Data: An Investigation of Diverse Signal Filtering Methods Within a General Linear Model Framework

**DOI:** 10.3389/fnhum.2020.00247

**Published:** 2020-07-14

**Authors:** Andrea Bizzego, Jan Paolo M. Balagtas, Gianluca Esposito

**Affiliations:** ^1^Department of Psychology and Cognitive Science, University of Trento, Trento, Italy; ^2^Psychology Program, School of Social Sciences, Nanyang Technological University, Singapore, Singapore; ^3^Lee Kong Chian School of Medicine, Nanyang Technological University, Singapore, Singapore

**Keywords:** fNIRS, signal processing, reproducibility, filtering, artifact removal, quality control

## 1. Introduction

We read with great interest the manuscript from Pinti et al. (2019), which aimed to shed a light on one of the main open topics in neuroimaging: the definition of reproducible and standardized pipelines for the preprocessing of functional Near InfraRed Spectroscopy (fNIRS) signals. In particular, Pinti and colleagues focused on the filtering step, evidencing a high heterogeneity of filter types adopted and settings that could undermine the reproducibility of the studies.

Thanks to technological progress, a new generation of fNIRS devices can be used to collect brain activity signals within diverse settings and contexts (e.g., multi-modal and multi-person experimental designs; Azhari et al., 2019, 2020) and for diverse applications (e.g., to study the dynamics of the human brain network; Vergotte et al., 2017). The proliferation of use cases and applications brings up the possibility of fragmentation of the knowledge, unless the scientific community begins to adopt rigorous and standardized methods to allow comparability and reproducibility of the findings.

## 2. fNIRS Data Processing Pipelines

The commented paper of Pinti et al. (2019) highlighted a high heterogeneity in the methods and settings adopted to filter fNIRS signals, which potentially could “lead to suboptimal papers or irreproducible studies and results.” To address this issue, they proposed a pre-processing procedure to aim at standardizing the filtering step.

Following the discussion initiated by Pinti et al. (2019), in this commentary we want to draw the attention on the heterogeneity of the processing sequences and their implementation. In fact, although critical, the filtering step addressed by Pinti et al. (2019), is only one step of the fNIRS data analysis procedure.

For the sake of simplicity, we restrict our discussion to the signal processing part: the steps that aim at removing the components of the signal that are not of interest for the study, typically noise, movement artifacts, and long-term physiological components. In other words, we excluded the different approaches that, starting from a processed fNIRS signal, aimed at obtaining statistical evidence to support the hypothesis of the study (e.g., statistical testing, statistical parametric mapping).

According to our experience, we can group the signal processing steps into four main categories:

- Quality control: procedures that aim to identify the signals that should be excluded from the subsequent analysis due to high magnitude of the noise (Orihuela-Espina et al., 2010). This step is usually done by computing signal quality metrics for each channel and setting threshold values to automatically reject those which do not meet the required standards (e.g., Cutini et al., 2014; Krampe et al., 2018; Lloyd-Fox et al., 2018). However, the choice of the appropriate set of signal metrics and thresholds might be arbitrary, or the decision on which channels to reject depends on a subjective evaluation upon visual inspections of the signals (Durantin et al., 2016; Holtzer et al., 2017).
- Artifact removal: procedures that aim to correct the portions of the signals that are affected by artifacts, where artifacts are a special type of noise components that are usually temporally and spatially localized, typically due to movements. Specific algorithms have been designed to scan each signal and identify the portions of the signals that contains artifacts (e.g., Lloyd-Fox et al., 2015; Pinti et al., 2015); as for the quality control, these algorithms rely on the appropriate definition of threshold values. In some cases, the portions with artifacts are manually validated (Holtzer et al., 2017).
- Noise removal: procedures that aim to improve the overall signal-noise-ratio. Typically, as highlighted by the commented paper of Pinti et al. (2019), these procedures are based on pass band filters (e.g., Santosa et al., 2017; Krampe et al., 2018; Lloyd-Fox et al., 2018), but there are also papers that use customized methods, for instance to remove long term trends or drifts (e.g., Chang et al., 2014; Pinti et al., 2015), or other types of filters (e.g., Cutini et al., 2014).
- Conversion: where the Lambert-Beer law is applied to convert the data from sensors to concentration of oxygenated and de-oxygenated hemoglobin. Note that this step can be done automatically by the acquisition device.

There is a high variability in how these steps are concatenated to compose the signal processing pipeline. To show this, we selected the 200 most cited papers found on Scopus with keywords “functional Near Infra-Red Spectroscopy OR fNIRS.” For the most recent 30 studies we identified and classified the signal processing steps of each paper according to the four categories above, and compared the different procedures adopted to process the fNIRS data (see Table 1). We decided to focus on the most recent studies to give an overview of the practices adopted in current research.

**Table 1:** Summary of the different sequences of signal processing steps adopted by the reviewed studies; sorted by number of steps and year of publication.

**References**	**Year**	**Pipeline**	**Manual step**
Lloyd-Fox et al. (2018)	2018	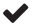 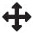 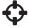 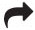 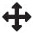	Yes
Holtzer et al. (2017)	2017	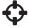 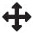 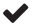 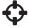 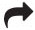	Yes
Pinti et al. (2015)	2015	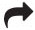 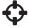 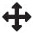 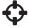	
Lloyd-Fox et al. (2015)	2015	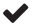 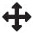 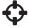 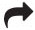	
Cutini et al. (2014)	2014	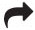 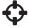 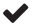 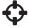	
Krampe et al. (2018)	2018	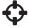 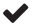 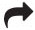	
Liu et al. (2015a)	2015	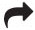 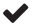 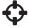	Yes
Kawasaki et al. (2015)	2015	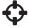 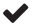 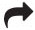	
Schecklmann et al. (2014)	2014	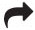 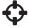 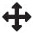	
Hirsch et al. (2017)	2017	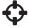 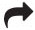	
Balardin et al. (2017)	2017	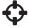 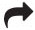	
Takeuchi et al. (2017)	2017	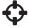 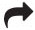	
Balconi et al. (2017)	2017	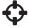 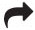	
Itakura et al. (2017)	2017	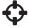 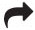	
Durantin et al. (2016)	2016	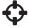 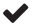	Yes
Kempny et al. (2016)	2016	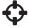 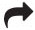	
Jiang et al. (2015)	2015	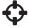 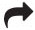	
Dempsey et al. (2015)	2015	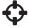 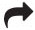	
Vanutelli and Balconi (2015)	2015	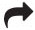 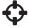	
Laguë-Beauvais et al. (2015)	2015	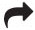 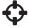	
Chang et al. (2014)	2014	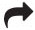 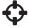	
Schaeffer et al. (2014)	2014	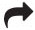 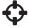	
Zhang and Khatami (2014)	2014	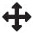 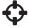	
Mandrick et al. (2013)	2013	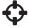 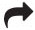	
Niu et al. (2013)	2013	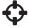 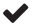	Yes
Santosa et al. (2017)	2017	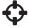	
Zhang et al. (2017)	2017	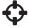	
Liu et al. (2015b)	2015	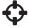	
Lin et al. (2014)	2014	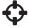	
Chuang and Sun (2014)	2014	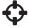	

*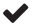: Quality control; 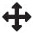: Artifacts removal; 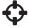: Noise removal; 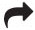: Conversion*.

We noted a high heterogeneity in the type of signal processing sequences, with the most common pattern being Conversion-Noise removal or Noise removal-Conversion. The processing pipeline of five studies involved a manual step, typically during the quality control, which was based on visual inspection and subjective evaluations. All steps were susceptible to heterogeneous solutions and customization, and were not limited to the “Noise removal” step.

## 3. Discussion

While it is important to discuss the single steps (as Pinti et al., 2019 did for the filtering step), it is also critical to pay attention to how these steps are concatenated and implemented.

Firstly, the order of execution of the different steps is important. For instance, some types of noise removal algorithms are affected by the presence of artifacts or noisy channels. Our proposal would be that the signal processing procedure follow this order: (i) Quality control: in order to remove the noisy channels that could eventually affect the result of the downstream analyses; (ii) Artifact removal: as localized artifacts may still influence the temporal and frequency characteristics of the signal and therefore affect the results of general algorithms in the downstream analysis; (iii) Noise removal: this step should not be influenced by highly noisy channels and artifacts, and thus should follow Quality control and Artifact removal; finally (iv): Conversion: this step can be also moved at the beginning of the sequence. However, it is important to ensure that the parameters adopted by the signal processing software have been calibrated on the appropriate signal (raw intensity, optical density, hemoglobin concentration).

Secondly, some steps (e.g., quality control) might involve the removal of some channels. Consequently, the number of independent signals used for statistical testing is, in general, different from the number of participants. We suggest to ensure that the actual number of signals is appropriate (e.g., through power analysis) and declare this number for each reported result.

Third: to maximize the impact of the research, we should favor, whenever possible, the adoption of quantitative methods and avoid proliferation of *ad-hoc* approaches. Any procedure that involves subjective decisions, as well as the application of customized algorithms, exposes the research to the risk of biased results. Additionally, *ad-hoc* solutions that worked well on another study might be inappropriate for use in other studies. Using algorithms provided by software applications for signal processing enables the standardization of processing stages. However, to be correctly applied, it is important to understand the algorithms, to select the appropriate processing parameters and to avoid artificial tuning to obtain outputs that comply with our expected results.

Last: in line with the commented paper of Pinti et al. (2019) in this commentary we focus mainly on the signal processing steps, but it is important to highlight that other aspects, not related to the signal processing, are also critical to ensure reliable results when using fNIRS data. The experimental protocols should be designed appropriately and considering the specific needs of fNIRS signal acquisition, for instance to avoid habituation effects or disengagement by the subjects (e.g., due to fatigue or boredom). The statistical methods that are applied on the metrics extracted from processed signal should be also appropriately defined, for instance identifying the correct control condition. It is critical to account for these aspects as they influence the signal processing pipeline itself and the reliability of the collected data.

As neuroscientists, we are convinced of the great potential of the fNIRS methodology, but as data scientists, we are also concerned that this high degree of customization of the signal processing procedures prevents the comparability between studies, their reproducibility and, ultimately, undermines the possibility of extracting new knowledge.

## Author Contributions

AB and GE conceived the study and wrote the manuscript. AB and JM reviewed the literature. All authors contributed to the article and approved the submitted version.

## Conflict of Interest

The authors declare that the research was conducted in the absence of any commercial or financial relationships that could be construed as a potential conflict of interest.

## References

[B1] Azhari A. Leck W. Gabrieli G. Bizzego A. Rigo P. Setoh P.. (2019). Parenting stress undermines mother-child brain-to-brain synchrony: a hyperscanning study. Sci. Rep. 9:11407. 10.1038/s41598-019-47810-4 31388049 PMC6684640

[B2] Azhari A. Lim M. Bizzego A. Gabrieli G. Bornstein M. H. Esposito G. (2020). Physical presence of spouse enhances brain-to-brain synchrony in co-parenting couples. Sci. Rep. 10:7569. 10.1038/s41598-020-63596-2 32371912 PMC7200679

[B3] Balardin J. B. Zimeo Morais G. A. Furucho R. A. Trambaiolli L. Vanzella P. Biazoli C. Jr. (2017). Imaging brain function with functional near-infrared spectroscopy in unconstrained environments. Front. Hum. Neurosci. 11:258. 10.3389/fnhum.2017.00258 28567011 PMC5434677

[B4] Balconi M. Pezard L. Nandrino J.-L. Vanutelli M. E. (2017). Two is better than one: the effects of strategic cooperation on intra-and inter-brain connectivity by fNIRS. PLoS ONE 12:e187652. 10.1371/journal.pone.0187652 29145416 PMC5690583

[B5] Chang P. H. Lee S.-H. Koo K.-M. Lee S.-H. Jin S.-H. Yeo S. S.. (2014). The cortical activation pattern by a rehabilitation robotic hand: a functional nirs study. Front. Hum. Neurosci. 8:49. 10.3389/fnhum.2014.00049 24570660 PMC3915242

[B6] Chuang C.-C. Sun C.-W. (2014). Gender-related effects of prefrontal cortex connectivity: a resting state functional optical tomography study. Biomed. Opt. Exp. 5, 2503–2516. 10.1364/BOE.5.002503 25136481 PMC4132984

[B7] Cutini S. Scarpa F. Scatturin P. Dell'Acqua R. Zorzi M. (2014). Number–space interactions in the human parietal cortex: enlightening the snarc effect with functional near-infrared spectroscopy. Cereb. Cortex 24, 444–451. 10.1093/cercor/bhs321 23081883

[B8] Dempsey J. P. Harris K. S. Shumway S. T. Kimball T. G. Herrera J. C. Dsauza C. M.. (2015). Functional near infrared spectroscopy as a potential biological assessment of addiction recovery: preliminary findings. Am. J. Drug Alcohol Abuse 41, 119–126. 10.3109/00952990.2014.983273 25588197

[B9] Durantin G. Scannella S. Gateau T. Delorme A. Dehais F. (2016). Processing functional near infrared spectroscopy signal with a kalman filter to assess working memory during simulated flight. Front. Hum. Neurosci. 9:707. 10.3389/fnhum.2015.00707 26834607 PMC4719469

[B10] Hirsch J. Zhang X. Noah J. A. Ono Y. (2017). Frontal temporal and parietal systems synchronize within and across brains during live eye-to-eye contact. Neuroimage 157, 314–330. 10.1016/j.neuroimage.2017.06.018 28619652 PMC5863547

[B11] Holtzer R. Schoen C. Demetriou E. Mahoney J. R. Izzetoglu M. Wang C.. (2017). Stress and gender effects on prefrontal cortex oxygenation levels assessed during single and dual-task walking conditions. Eur. J. Neurosci. 45, 660–670. 10.1111/ejn.13518 28028863 PMC5339034

[B12] Itakura M. Pu S. Ohdachi H. Matsumura H. Yokoyama K. Nagata I.. (2017). Association between social functioning and prefrontal cortex function during a verbal fluency task in schizophrenia: a near-infrared spectroscopic study. Psychiatry Clin. Neurosci. 71, 769–779. 10.1111/pcn.12548 28657683

[B13] Jiang J. Chen C. Dai B. Shi G. Ding G. Liu L.. (2015). Leader emergence through interpersonal neural synchronization. Proc. Natl. Acad. Sci. U.S.A. 112, 4274–4279. 10.1073/pnas.1422930112 25831535 PMC4394311

[B14] Kawasaki S. Nishimura Y. Takizawa R. Koike S. Kinoshita A. Satomura Y.. (2015). Using social epidemiology and neuroscience to explore the relationship between job stress and frontotemporal cortex activity among workers. Soc. Neurosci. 10, 230–242. 10.1080/17470919.2014.997370 25580832

[B15] Kempny A.M. James L. Yelden K. Duport S. Farmer S. Playford E. D.. (2016). Functional near infrared spectroscopy as a probe of brain function in people with prolonged disorders of consciousness. NeuroImage 12, 312–319. 10.1016/j.nicl.2016.07.013 27547728 PMC4983150

[B16] Krampe C. Strelow E. Haas A. Kenning P. (2018). The application of mobile fnirs to “shopper neuroscience”–first insights from a merchandising communication study. Eur. J. Market. 52, 244–259. 10.1108/EJM-12-2016-0727

[B17] Laguë-Beauvais M. Fraser S. A. Desjardins-Crépeau L. Castonguay N. Desjardins M. Lesage F.. (2015). Shedding light on the effect of priority instructions during dual-task performance in younger and older adults: a fNIRS study. Brain Cogn. 98, 1–14. 10.1016/j.bandc.2015.05.001 26046834

[B18] Lin Z.-J. Li L. Cazzell M. Liu H. (2014). Atlas-guided volumetric diffuse optical tomography enhanced by generalized linear model analysis to image risk decision-making responses in young adults. Hum. Brain Mapp. 35, 4249–4266. 10.1002/hbm.22459 24619964 PMC4282392

[B19] Liu N. Cui X. Bryant D. M. Glover G. H. Reiss A. L. (2015a). Inferring deep-brain activity from cortical activity using functional near-infrared spectroscopy. Biomed. Opt. Exp. 6, 1074–1089. 10.1364/BOE.6.001074 25798327 PMC4361422

[B20] Liu T. Saito H. Oi M. (2015b). Role of the right inferior frontal gyrus in turn-based cooperation and competition: a near-infrared spectroscopy study. Brain Cogn. 99, 17–23. 10.1016/j.bandc.2015.07.001 26189111

[B21] Lloyd-Fox S. Blasi A. Pasco G. Gliga T. Jones E. J. Murphy D.. (2018). Cortical responses before 6 months of life associate with later autism. Eur. J. Neurosci. 47, 736–749. 10.1111/ejn.13757 29057543 PMC5900943

[B22] Lloyd-Fox S. Wu R. Richards J. E. Elwell C. E. Johnson M. H. (2015). Cortical activation to action perception is associated with action production abilities in young infants. Cereb. Cortex 25, 289–297. 10.1093/cercor/bht207 23975948 PMC4303799

[B23] Mandrick K. Derosiere G. Dray G. Coulon D. Micallef J.-P. Perrey S. (2013). Prefrontal cortex activity during motor tasks with additional mental load requiring attentional demand: a near-infrared spectroscopy study. Neurosci. Res. 76, 156–162. 10.1016/j.neures.2013.04.006 23665138

[B24] Niu H. Li Z. Liao X. Wang J. Zhao T. Shu N.. (2013). Test-retest reliability of graph metrics in functional brain networks: a resting-state fNIRS study. PLoS ONE 8:e72425. 10.1371/journal.pone.0072425 24039763 PMC3767699

[B25] Orihuela-Espina F. Leff D. R. James D. R. Darzi A. W. Yang G.-Z. (2010). Quality control and assurance in functional near infrared spectroscopy (fnirs) experimentation. Phys. Med. Biol. 55:3701. 10.1088/0031-9155/55/13/009 20530852

[B26] Pinti P. Aichelburg C. Lind F. Power S. Swingler E. Merla A.. (2015). Using fiberless, wearable fnirs to monitor brain activity in real-world cognitive tasks. J. Visual. Exp. 106:e53336. 10.3791/53336 26651025 PMC4692764

[B27] Pinti P. Scholkmann F. Hamilton A. Burgess P. Tachtsidis I. (2019). Current status and issues regarding pre-processing of fNIRS neuroimaging data: an investigation of diverse signal filtering methods within a general linear model framework. Front. Hum. Neurosci. 12:505. 10.3389/fnhum.2018.00505 30687038 PMC6336925

[B28] Santosa H. Aarabi A. Perlman S. B. Huppert T. (2017). Characterization and correction of the false-discovery rates in resting state connectivity using functional near-infrared spectroscopy. J. Biomed. Opt. 22:055002. 10.1117/1.JBO.22.5.055002 28492852 PMC5424771

[B29] Schaeffer J. D. Yennu A. S. Gandy K. C. Tian F. Liu H. Park H. (2014). An fNIRS investigation of associative recognition in the prefrontal cortex with a rapid event-related design. J. Neurosci. Methods 235, 308–315. 10.1016/j.jneumeth.2014.07.011 25063422

[B30] Schecklmann M. Giani A. Tupak S. Langguth B. Raab V. Polak T.. (2014). Functional near-infrared spectroscopy to probe state-and trait-like conditions in chronic tinnitus: a proof-of-principle study. Neural Plast. 2014:894203. 10.1155/2014/894203 25478237 PMC4248328

[B31] Takeuchi N. Mori T. Suzukamo Y. Izumi S.-I. (2017). Integration of teaching processes and learning assessment in the prefrontal cortex during a video game teaching?learning task. Front. Psychol. 7:2052. 10.3389/fpsyg.2016.02052 28119650 PMC5220187

[B32] Vanutelli M. E. Balconi M. (2015). Perceiving emotions in human?human and human?animal interactions: hemodynamic prefrontal activity (fnirs) and empathic concern. Neurosci. Lett. 605, 1–6. 10.1016/j.neulet.2015.07.020 26272301

[B33] Vergotte G. Torre K. Chirumamilla V. C. Anwar A. R. Groppa S. Perrey S.. (2017). Dynamics of the human brain network revealed by time-frequency effective connectivity in fnirs. Biomed. Opt. Exp. 8, 5326–5341. 10.1364/BOE.8.005326 29188123 PMC5695973

[B34] Zhang M. Liu T. Pelowski M. Jia H. Yu D. (2017). Social risky decision-making reveals gender differences in the tpj: a hyperscanning study using functional near-infrared spectroscopy. Brain Cogn. 119, 54–63. 10.1016/j.bandc.2017.08.008 28889923

[B35] Zhang Z. Khatami R. (2014). Predominant endothelial vasomotor activity during human sleep: a near-infrared spectroscopy study. Eur. J. Neurosci. 40, 3396–3404. 10.1111/ejn.12702 25156240

